# Histopathological Findings after Descemet's Stripping Automated Endothelial Keratoplasty for the Management of Descemet's Membrane Breaks Secondary to Obstetrical Forceps Injury

**DOI:** 10.1155/2012/474795

**Published:** 2012-06-28

**Authors:** Luis J. Haddock, Sander R. Dubovy, Victor L. Perez

**Affiliations:** Department of Ophthalmology, Bascom Palmer Eye Institute, University of Miami Miller School of Medicine, 900 NW 17th St., Miami, FL 33136, USA

## Abstract

Case of a 39 y/o male patient that presented due to decreased vision and pain in the left eye secondary to corneal edema related to vertical Descemet's membrane breaks. The patient's past medical history was remarkable for a complicated vaginal delivery with the use of obstetrical forceps and presumed obstetrical forceps corneal injury. Herein, we demonstrate for the first time the use of descemet's stripping automated endothelial keratoplasty (DSAEK) in the management of this complication and for the first time show histologically the area of prior descemet's membrane break in the submitted stripped descemet's membrane.

## 1. Introduction

Descemet's membrane breaks occur secondary to a variety of conditions that include congenital glaucoma, keratoconus, and trauma. These breaks are classified depending on their time of presentation in early childhood or adulthood, laterality, affecting one or both eyes, and corneal orientation, as vertical, horizontal, or oblique. Descemet's breaks can be seen in newborns after complicated forceps delivery because Descemet's membrane is thin and susceptible to stretching at birth [1]. The vertical breaks result from a horizontal stretching of the globe that occurs with vertical compression of the eye between the orbital roof and the blade of the obstetric forceps [2]. These patients can present with decreased vision early in life, secondary to corneal opacification, induced astigmatism, and/or amblyopia, or in adulthood secondary to corneal edema resulting from gradual endothelial decompensation of a previously compromised endothelium [3]. Here we report the clinical history and histopathological correlation of the findings in the stripped Descemet's membrane of a patient who underwent Descemet's stripping endothelial keratoplasty to correct a cornea that failed because of vertical Descemet's breaks associated to forceps injury during delivery.

## 2. Case Report

A 39 y/o male patient presented with a 2-month history of decreased vision, halos, pain, and photophobia of the left eye. The patient was diagnosed with keratoconus at age 16, for which he used rigid contact lens in the left eye, with a best corrected visual acuity (VA) of 20/60. The patient's past medical history revealed that he had a complicated vaginal delivery with the use of obstetrical forceps. 

**Figure 1:**
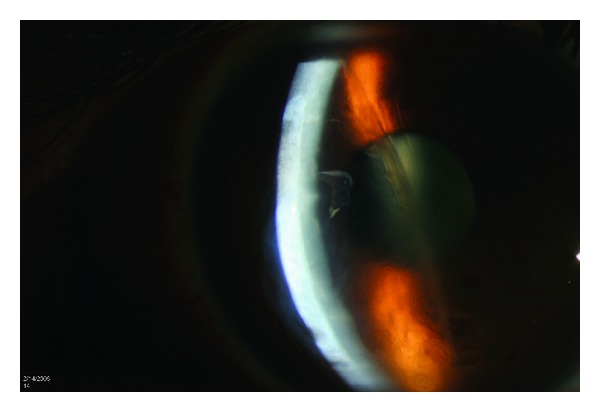
Slit lamp photo showing corneal edema and vertical opaque lines at the level of Descemet's membrane.

Clinical examination showed a VA of 20/400 in the left eye with a refraction of −4.00 + 5.00 × 095 and a stable VA of 20/20 in the right eye with a refraction of −1.00 + 0.50 × 180. Slit lamp biomicroscopy displayed corneal stromal and epithelial edema associated with centrally located parallel vertical opaque lines at the level of Descemet's membrane (Figure 1). The right eye had no corneal changes and the intraocular pressure and the posterior segment examination was unremarkable in both eyes. Further studies included a corneal topography (Orbscan) that ruled out keratoconus and showed regular astigmatism of 5.7 D at an axis (097°) that correlated with the location of the striae on slit lamp, and an anterior segment OCT that showed hypereflective linear structures protruding into the anterior chamber at the level of the posterior cornea.

**Figure 2:**
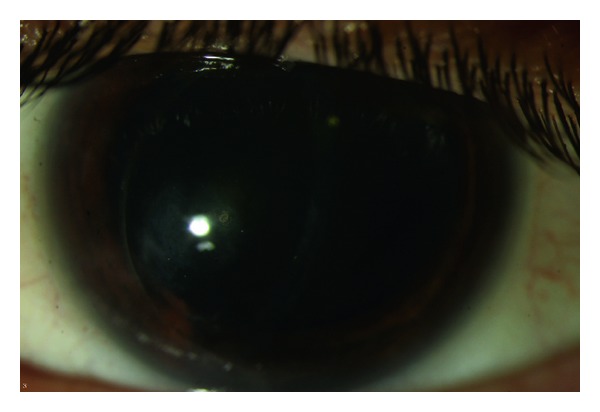
Slit lamp biomicroscopy photo at 1.5 years after DSAEK showing a nonedematous cornea with minimal superficial scarring.

A diagnosis of corneal edema secondary to endothelial decompensation in the left eye secondary to forceps injury and history of Descemet's membrane break was made. The patient underwent a Descemet's stripping automated endothelial keratoplasty (DSAEK) in order to replace the diseased posterior corneal lamella that included a Descemet's membrane previously traumatized by the obstetrical forceps, and an endothelium that had undergone gradual decompensation. The stripped Descemet's membrane was submitted for histopathological evaluation. The patient had good visual outcome after DSAEK with BCVA of 20/80. Slit lamp examination revealed a well appositioned graft, and a clear cornea with minimal superficial scarring (Figure 2). Mild superficial corneal scarring and preexisting amblyopia limited final visual acuity.

**Figure 3:**
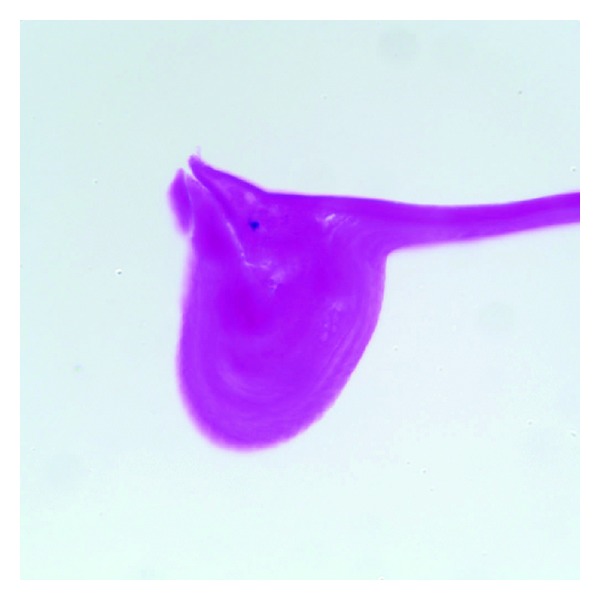
Histopathology of the stripped Descemet's membrane demonstrating an area of nodular thickening of Descemet's membrane composed of concentric scrolls of PAS-positive material (periodic acid-schiff stain, original magnification x400).

Histological examination of the stripped Descemet's membrane revealed endothelial attenuation and a thickened PAS-positive membrane with areas of nodular thickening at the edge of the initial break composed of concentric deposits of PAS-positive material (Figure 3). 

## 3. Discussion

The clinical history and histopathological findings in the stripped Descemet's membrane of a 39 y/o patient that underwent DSAEK to treat an endothelial decompensation associated to vertical Descemet's membrane breaks that occurred after obstetrical forceps delivery are reported. The initial Descemet's membrane rupture causes an acute corneal edema shortly after birth that typically clears over the next few weeks at which time vertical or oblique striae are seen clinically representing permanent linear thickening of Descemet's membrane at areas of prior breaks [4]. These striae may cause decreased vision secondary to induce astigmatism, high myopia, glare, visual opacification, and amblyopia. In this case, the patient had 5 cylinders of astigmatism with an axis corresponding to the axis of the break that is similar to previous reports that described a mean cylinder of astigmatism of 6.9 D (3.0–10.50 D) in the affected eye compared to 0.36 D (0.0–1.50 D) in the noninvolved eye, with a steep axis parallel to that of the breaks [4]. Patients can also present many years later with corneal edema resulting from endothelial decompensation of a previously compromised endothelium. The patient reported presented with corneal edema secondary to endothelial decompensation at age 39, which correlates with the reported mean time of 37 years (range: 25–44) for clinically significant endothelial decompensation after the initial injury [5, 6].

Honig et al. [5] in 1996, classified the histological findings of Descemet's breaks in transplanted corneal specimens secondary to forceps injury into four categories: type 1 had a scroll at one margin and a fragment of Descemet's membrane extending into the anterior chamber at the other margin; type 2 had scrolls of Descemet's membrane at both margins of the tear; type 3 had fibrous proliferation around the area of break creating a retrocorneal membrane; type 4 contained a small discontinuity in Descemet's membrane with minimal fibrosis. This case resembles a type 1 injury as we noted a scroll of Descemet's membrane at one margin of the break on the submitted Descemet's membrane after DSAEK. The histological finding of scrolls and nodular thickening of Descemet's membrane seen at the edge of the original breaks are a result of a healing response from the corneal endothelium that laid down many layers of new basement membrane in order to cover the defect [5, 7]. These findings correlate clinically with the striae and ridges noted on exam. 

In this paper we demonstrate the use of DSAEK in the management of corneal breaks secondary to forceps injury and show that surgery was successful in removing the affected area of Descemet's membrane as seen in Figure 3. In addition, for the first time we demonstrate that that this type of injury can be diagnosed histopathologically many years later, with the submission of the striped Descemet's membrane at the time of DSAEK.

## References

[1] Jain IS, Singh YP, Grupta SL, Gupta A (1980). Ocular hazards during birth. *Journal of Pediatric Ophthalmology and Strabismus*.

[2] Krachmer JH, Mannis MJ, Holland EJ (2005). *Cornea*.

[3] Angell LK, Robb RM, Berson FG (1981). Visual prognosis in patients with ruptures in Descemet’s membrane due to forceps injuries. *Archives of Ophthalmology*.

[4] Ponchel C, Malecaze F, Arné JL, Fournié P (2009). Descemet stripping automated endothelial keratoplasty in a child with descemet membrane breaks after forceps delivery. *Cornea*.

[5] Honig MA, Barraquer J, Perry HD, Riquelme JL, Green WR (1996). Forceps and vacuum injuries to the cornea: histopathologic features of twelve cases and review of the literature. *Cornea*.

[6] Spencer WH, Fergunson WJ, Shaffer RN, Fine M (1966). Late degenerative changes in the cornea following breaks in Descemet’s membrane. *Transactions of the American Academy of Ophthalmology and Otolaryngology*.

[7] Tetsumoto K, Kubota T, Rummelt V, Holbach LM, Naumann GOH (1993). Epithelial transformation of the corneal endothelium in forceps birth-injury-associated keratopathy. *Cornea*.

